# The Role of miRNAs in Extracellular Matrix Repair and Chronic Fibrotic Lung Diseases

**DOI:** 10.3390/cells10071706

**Published:** 2021-07-06

**Authors:** Kauna Usman, Aileen Hsieh, Tillie-Louise Hackett

**Affiliations:** 1 Centre for Heart Lung Innovation, St. Paul’s Hospital, Vancouver, BC V6Z 1Y6, Canada; Kauna.Usman@hli.ubc.ca (K.U.); Aileen.Hsieh@hli.ubc.ca (A.H.); 2 Department of Anesthesiology, Pharmacology and Therapeutics, University of British Columbia, Vancouver, BC V5X 1M9, Canada

**Keywords:** extracellular matrix, lung, miRNA, asthma, chronic obstructive pulmonary disease, idiopathic pulmonary disease

## Abstract

The lung extracellular matrix (ECM) plays a key role in the normal architecture of the lung, from embryonic lung development to mechanical stability and elastic recoil of the breathing adult lung. The lung ECM can modulate the biophysical environment of cells through ECM stiffness, porosity, topography and insolubility. In a reciprocal interaction, lung ECM dynamics result from the synthesis, degradation and organization of ECM components by the surrounding structural and immune cells. Repeated lung injury and repair can trigger a vicious cycle of aberrant ECM protein deposition, accompanied by elevated ECM stiffness, which has a lasting effect on cell and tissue function. The processes governing the resolution of injury repair are regulated by several pathways; however, in chronic lung diseases such as asthma, chronic obstructive pulmonary disease (COPD) and idiopathic pulmonary disease (IPF) these processes are compromised, resulting in impaired cell function and ECM remodeling. Current estimates show that more than 60% of the human coding transcripts are regulated by miRNAs. miRNAs are small non-coding RNAs that regulate gene expressions and modulate cellular functions. This review is focused on the current knowledge of miRNAs in regulating ECM synthesis, degradation and topography by cells and their dysregulation in asthma, COPD and IPF.

## 1. Introduction

The lung extracellular matrix (ECM) plays a key role in the normal architecture of the lung, from embryonic lung development, alveolarization at birth, to mechanical stability and elastic recoil of the breathing adult lung [1,2]. The lung ECM provides both biochemical and biophysical cues, which direct cellular functions and differentiation. The lung ECM can also alter the biochemical environment of surrounding cells, by storing and sequestering growth factors and cytokines to regulate spatially and temporally their bioavailability. The lung ECM can modulate the biophysical environment of cells through ECM stiffness, porosity, topography (spatial arrangement and orientation) and insolubility. Lung ECM molecules connect to cells via integrins, syndecans and other receptors to influence cell signalling, migration and proliferation. In response, the lung ECM is remodelled by cells, whereby its components are deposited, degraded or modified in a reciprocal relationship [3,4]. The objective of this review is to evaluate the current knowledge on the regulation of the lung ECM in health and disease by cells through the epigenetic regulation by miRNAs.

## 2. The Lung Extracellular Matrix

The “core matrisome” of the lung ECM comprises over 300 proteins [5], and each has distinct physical and biochemical properties that dictate its function. The lung matrisome is organized into two main structural subtypes: (1) basement membranes that provide the anchorage site for epithelia, endothelia, muscle, fat and peripheral nerves; and (2) the interstitial matrix that connects the structural cells within the lung. The predominant components of the basement membrane include collagen IV, collagen V, laminins (which are also the most abundant non-collagenous component), chondroitin sulphate proteoglycans (perlecan, agrin and dystroglycan), entactin, fibronectin, fibulin I and fibulin II [6,7]. The interstitial matrix comprises largely a meshwork of elastin, collagen I, collagen III, fibronectin, vitronectin, tenascin, versican and decorin [8,9]. The matrisome network of protein–protein and protein–proteoglycan interactions form the supramolecular assemblies of collagen and elastic fibers that shape the structural scaffold of the ECM within the lung. The arrangement of this meshwork allows for the non-linear stress–strain behaviour within the lung, with elastin providing viscoelasticity and collagen tensile strength. 

## 3. How Cells Modify the Lung Extracellular Matrix

The lung ECM functions as a physical barrier, an anchorage site and a migration highway for cells within the organ. The ECM dynamics within each cellular niche are tightly regulated to ensure normal development, physiology and homeostasis of organ systems. Alterations in the lung ECM environment result from the synthesis, degradation or altered organization of ECM components by surrounding structural and immune cells. This is achieved by redundancy in the mechanisms used to modulate the expression and function of the ECM and ECM-modifying enzymes. When such control mechanisms are corrupted, ECM dynamics become deregulated, leading to various congenital defects or disease.

### 3.1. Synthesis of ECM by Cells

There is a dynamic biophysical and biochemical reciprocal interaction between cells and their surrounding ECM microenvironment. Variations in cell phenotype determine the assembly and composition of ECM proteins, resulting in different tissue morphologies and functions. To initiate ECM production, cells relay signals via the membrane and cytoskeleton by recruiting factors such as cytokines, adhesion molecules and growth factors to activate gene transcription. The synthesis and turnover expression rates of different ECM proteins within a specific tissue vary. For example, in the normal lung, collagen synthesis is estimated to be 10% per day, while that of other non-collagenous proteins is up to 35% per day [10,11]. Interestingly, the rate of ECM synthesis in a specific tissue may also vary as a result of age-related changes. Mays and colleagues showed that lung collagen synthesis decreased from 13% per day in a one-month-old mouse to 0.97% per day in two-year-old mice [12].

Mesenchymal cells are the dominant producers of ECM proteins [13,14], with fibroblasts being the largest synthesizer of the fibrillar ECM components such as collagen, laminins and fibronectin [15]. Basement membrane proteins such as collagen IV and laminins are synthesized by epithelial and endothelial cells. Other specialized cells involved in the synthesis of ECM protein include smooth muscle cells (versican), vascular smooth muscle cells and endothelial cells (Emilin-1 and 2). Inflammatory cells including monocytes and macrophages are also able to synthesize proteoglycans such as perlecan, serglycin, syndecans, amyloid precursor-like protein-2 and glypican-1 in response to anti-inflammatory signals [16]. In a physiological state, once ECM protein expression turnover is achieved, the activated cells return to a quiescent state to prevent the exaggerated synthesis of ECM proteins [17,18]. 

### 3.2. Degradation of ECM by Cells

ECM degradation is an essential process for tissue homeostasis and can be detrimental in tissue remodeling. The most significant enzymes in ECM remodelling are metalloproteinases [19], which include two main families of metalloproteinases, matrix metalloproteinases (MMP) and A disintegrin and metalloproteinase with thrombospondin motifs (ADAMTS) families.

MMPs are grouped according to their modular domain structures. Presently, they are classified into eight groups: minimal domain (MMP-7, MMP-26), simple hemopexin domain (MMP-1, MMP-3, MMP-8, MMP-10, MMP-12, MMP-13, MMP-18, MMP-19, MMP-20, MMP-22, MMP-27), gelatin-binding (MMP-2, MMP-9), membrane-type (MMP-14, MMP-15, MMP-16, MMP-24), furin-activated secreted (MMP-11, MMP-28), glycophosphatidyl inositol-anchoring domain (MMP-17, MMP-25), vitronectin-like insert linker-less (MMP-23) and cysteine/proline-rich interleukin-1 receptor-like domain MMPs (MMP-23) [20]. All MMPs contain a catalytic domain and an auto-inhibitory prodomain, which houses an active zinc-binding site. The unique catalytic domains of each MMP dictate the substrate recognition and cleavage site. For example, both MMP-2 and MMP-9 have a high affinity towards collagen and other ECM proteins including elastin, fibronectin and vitronectin, whereas MMP-3 and MMP-10 have a higher affinity towards proteoglycans, laminins and fibronectin [19,21]. The auto-inhibitory prodomains can be activated through direct cleavage by other endoproteinases, allosteric conformation or modification by reactive oxygen species and non-physiological agents [22]. Once activated, MMPs can mediate ECM protein degradation by directly cleaving the ECM substrate precursor proteins. Additionally, MMPs can modify the activities of signalling molecules such as cell surface receptors, adhesion molecules, chemokines, growth factors and proteinase inhibitors within the ECM, enabling modification of intercellular junctions and cell–ECM adhesions [23]. For example, MMP-9 can alter epithelial migration through transforming growth factor (TGF)-β activation from an inactive fibronectin-TGF-β complex [24]. Alternatively, MMPs can modulate signalling molecules by generating cleavage fragments with cell-binding properties, from the cleaved substrate. For example, tumstatin, a cleaved fragment of the alpha-subunit of collagen IV by MMP-9, has proapoptotic properties and also serves as an angiostatic peptide [25]. Another example is TGF-β; in its latent state it is bound to the fibrillin protein latency-associated peptide (LAP), a substrate of MMP-2, MMP-9, MMP-13 and MMP-14. Cleavage of this complex by these MMPs releases the TGF-β and increases its availability for signalling [22].

Similarly, ADAMTS proteinases have a prodomain amongst several other domains including the disintegrin and metalloproteinase domains. ADAMTS (except ADAMTS-2) have a high affinity towards degrading proteoglycans; hence, they are often called proteoglycanases. For example, the proteoglycan aggrecan is targeted for degradation by ADAMTS-4 and ADAMTS-5 [26]. Serine proteases (elastase and cathepsin G) and cysteine proteases (cathepsins B and K) are also ECM-degrading enzymes secreted outside the cell to digest the ECM [27]. Serine proteases play various roles in ECM degradation and target essentially all the ECM components. As an example, Albrengues and colleagues demonstrated that laminin-111 is sequentially cleaved by neutrophil elastase and MMP-9 [28]. Cathepsin K has potent aggrecan degrading activity that further potentiates degradation of collagen I and collagen II [29].

Dysregulation of ECM protein degradation can have significant effects on the host tissue architecture. Therefore, proteases are controlled by their endogenous activators and inhibitors. MMPs and ADAMTS are tightly controlled through their transcription, activation and inactivation by tissue inhibitor of metalloproteinases (TIMPs 1, 2, 3 and 4). TIMPs have different binding affinities, for example, TIMP-3 has a high affinity towards inhibiting ADAMTS-4 and ADAMTS-5 [30], while TIMP-1 is more potent for MMP-3 and MMP-7(22). Other non-specific protease inhibitors such as alpha-2 macroglobulin can also inhibit MMPs [22], while plasminogen activator inhibitor-1 and alpha-2 antiplasmin target serine proteases [31].

### 3.3. Alterations in ECM Topography by Cells

The cross-linking of ECM proteins via covalent and noncovalent modifications greatly influences the organization and topography of the ECM environment, which in turn has fundamental effects on cell behavior [32,33,34]. Proteomic studies in mouse models have shown that during the various stages of lung development and physiological processes, the ECM undergoes large changes in its topography and composition [35]. In addition, the composition of the matrix network and architectural changes in the ECM in varying physiological states has also been documented [36]. Interstitial collagens can undergo several posttranslational modifications, including covalent and noncovalent cross-linking. The extent of collagen–elastin intermolecular crosslinking is largely determined by the expression and activation of the family of lysyl oxidases (LOX) and lysyl hydroxylases. For example, up-regulation of collagen cross-linking, due to excess LOX activity, leads to an increase in the matrix stiffness and thus tensile strength of tissues, which can profoundly influence cellular behaviours [15,37]. Inhibition of LOX has also been shown to decrease fibril collagen thickness and subsequent tissue stiffening in IPF [38]. Levental and colleagues showed that collagen crosslinking can modulate breast tissue fibrosis and stiffness, leading to enhanced growth factor signalling and breast malignancies [37].

In the pulmonary vasculature, Emlinin-1 and -2 (elastin microfibril interface-located proteins) are incorporated into elastin and fibrillin microfibrils and have been shown to be important extracellular regulators of global TGF- β, BNP (bone morphogenic protein) and Wnt signaling pathways [39]. Alteration in the expression of Emilin-1 and -2 results in disruption of cell adhesion, apoptosis, migration and proliferation and destabilization of microfibrils, which subsequently alters the ECM architecture and tissue integrity [39,40,41,42,43,44].

Cells can also manipulate ECM tomography through traction of ECM proteins by binding of cell receptors and anchorage with the cell cytoskeleton. Such traction force interferes with the cross-linking bonds between ECM proteins and the cell’s actin cytoskeleton, leading to modification of cellular signalling and gene expression. For example, stretching of fibronectin by cellular traction force increases its binding force to integrin receptors, other fibronectin dimers and collagen. This, in turn, increases the size, density and rigidity of fibronectin fibres and causes pleiotropic changes in cell growth, differentiation and migration [45], often influencing the progression of fibrosis in cells.

In summary, ECM synthesis, degradation and topography are controlled at multiple levels including transcriptional and posttranslational regulation. For the remainder of this review, we will assess the role of epigenetic regulation through the expression and release of miRNAs by cells and their influence on the lung ECM microenvironment in health and disease.

## 4. The Role of miRNAs in Modifying the ECM Microenvironment

**Figure 1:**
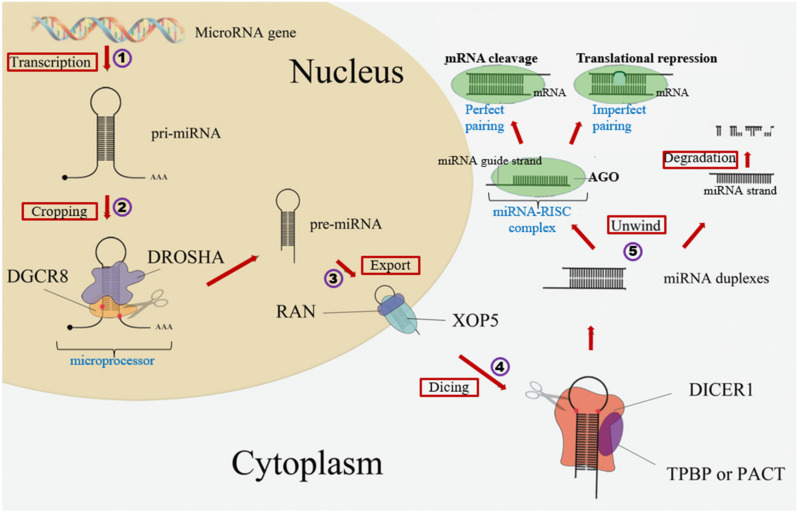
miRNA biogenesis. RNA genes are (1) transcribed to primary miRNA (pri-miRNA) by RNA polymerase enzyme II and then later (2) cropped by the microprocessor (DROSHA-DGCR8) to precursor miRNA (pre-miRNA). (3) Export of these pre-miRNA from the nucleus into the cytoplasm by Exportin-5 (XPO5). (4) Dicing by DICER and then (5) processing into mature miRNA and loaded on the RNA-inducing silencing complex (RISC) to mediate various functions. This figure is adapted with permission from He J. et al, PeerJ, 2016 (https://doi.org/10.7717/peerj.2706). (Accessed on 10 May 2021) DGCR8—DiGeorge syndrome critical region 8, MicroRNA—miRNA, DROSHA—Ribonuclease III, RAN—Ras-related nuclear protein, XOP5—Exportin 5, DICER1—Riboendonuclease, TPBP—Transactivation response RNA binding protein, PACT—Protein activator, AGO—Argonaute protein, mRNA—messenger RNA.

Current estimates show that more than 60% of the human coding transcripts are regulated by miRNAs [46]. Rodrigues et al. (2004) and Griffiths-Jones et al. (2006) have listed 2588 annotated miRNAs in the human genome [47,48]. MiRNAs are short, non-coding RNAs that are approximately 22 nucleotides long. As shown in Figure 1, miRNAs are primarily generated via (1) transcription by RNA polymerase enzyme, after which they undergo (2) cropping to precursor miRNAs (pre-miRNA) by the microprocessor complex of DROSHA (ribonuclease III Drosha and DGCR8 (DiGeorge Syndrome Critical Region 8)). The precursor miRNAs are then (3) exported by the nuclear protein exportin-5 (XPO5) from the cell nucleus into the cytoplasm where they are further processed to mature miRNA following (4) dicing by RNase III endonuclease Dicer. The functional strand of the mature miRNA is then (5) loaded into the risk inducing silencing complex (RISC) comprising the argonaute (AGO) family of proteins to mediate various functions [49].

**Figure 2:**
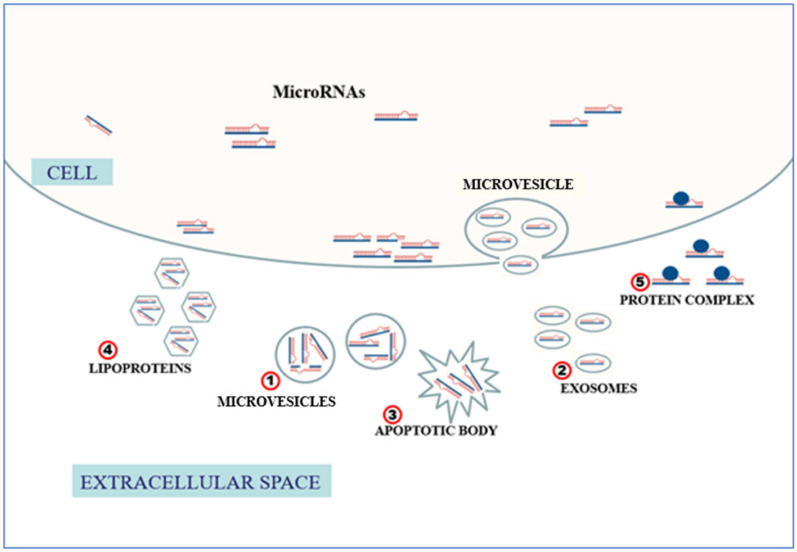
Release of miRNA to the extracellular compartment. Mature miRNAs are transported out of the cell by (1) enwrapping into microvesicles, (2) selective incorporation into exosomes, (3) apoptotic bodies, (4) coupling with high-density lipoproteins and (5) protein complexes such as AGO to be released into the extracellular environment or other cells.

The classic mechanism of miRNA-mediated gene regulation involves binding of the miRNA seed sequence to the 3’UTR of mRNA and, to a lesser extent, the 5’UTR of the mRNA, gene promoters or coding sequences [50]. Based on the level of complementarity binding with the interacting sites, they trigger transcript degradation or inhibition of translation. Until recently, it was believed that miRNAs function within their cell of origin. However, there is now strong evidence for miRNAs in the extracellular microenvironment [51,52,53,54,55] and their involvement in cell–cell communication [56,57]. Recent studies have shown that miRNAs are present in the extracellular environment, including different biological fluids such as bronchial lavage and breast milk [53]. As shown in Figure 2, the release of miRNA into the extracellular compartment is mediated by (1) enwrapping into microvesicles, (2) selective incorporation into exosomes (3) or apoptotic bodies, and (4) coupling with high-density lipoproteins or (5) protein complexes such as AGO to enable them to function as intercellular signalling molecules [58,59,60,61].

It is now understood that some miRNAs are exclusively associated with microvesicles such as let-7a, and other miRNAs act independently of vesicles such as miR-122 [62]. The stability of AGO proteins, and the remarkable degree of impermeability of microvesicles and exosomes to RNases, means long-term stability of miRNAs in vesicles. The miRNA pathway, through extracellular release thus possess a critical mechanism of regulating the ECM environment [63].

## 5. ECM Regulation by miRNAs

MiRNAs are involved in a number of cellular processes that regulate the synthesis of ECM molecules in various organs and model systems. On 14 May 2021, we searched the scientific literature in PubMed (with no date or language restrictions) for the following search terms “each ECM protein” and “miRNA”. As shown in Table 1, for 19 ECM proteins expressed within the lung, all have been shown in one or more model system to be regulated by the classic regulatory mechanism exerted by miRNAs, by direct interaction with their cognate binding mRNA. Specifically, we found 35 miRNAs that have been shown to regulate ECM protein translation through binding the 3’UTR. For example, down-regulation of miR-206 modulates in human lung adenocarcinoma epithelial cell line (A549’s) increase in the translation of fibronectin 1 through direct binding to the 3’UTR of the fibronectin 1 gene [64].

A shown in Table 1, we found 41 miRNAs that have been shown to indirectly regulate the ECM by targeting ECM transcription factors including transcriptional activators and repressors. For example, in skin fibroblasts, TGF-β1/smad-induced collagen 1 synthesis is enhanced due to up-regulation of heat shock protein 47 (HSP47), a chaperon protein associated with collagen 1 synthesis and down-regulation of miR-29b expression [65]. Suppression of HSP90 by over-expressing miR-628-3p via its 3’UTR and miR-27 via Akt signaling in A549 lung cancer cells and esophageal squamous cell carcinoma, respectively, has been shown to modulate collagen deposition [66,67,68]. The relationship between ECM proteins and miRNA is not always unidirectional, as numerous downstream signalling pathways can also be affected. For example, in breast cancer cells, miR-10b binds with the 3’UTR of the epithelial transmembrane proteoglycan syndecan-1, leading to down-regulated expression. However, the down-regulation of syndecan-1 by miR-10b results in the increased expression of fibronectin and laminin via the focal adhesion kinase and Rho-GTPase pathways [69].

MiRNAs are also able to influence ECM expression through protein degradation. To date, 23 miRNAs have been shown to affect ECM composition through the regulation of proteases and anti-proteinases. For example, the expressions of MMP-9, MMP-16 and TIMP-3 associated with collagen homeostasis, cell migration and invasion are modulated by miR-373, miR-146b and miR-21, respectively [70,71,72].

Various cytokines and growth factors such as TGF-β and epidermal growth factors play important roles in the modulation of ECM assembly, and most of these functions are regulated by miRNAs. Fifteen miRNAs have been shown to influence ECM expression through growth factors and cytokines expression. Specifically, MiR-1, miR-31 and miR-206 have been shown to regulate lung fibroblast cells, a key player in the synthesis, deposition and remodelling of ECM through the vascular endothelial growth factor (VEGF), chemokine (c-c motif) ligand (CCL)-2 and FoxO3a signalling pathways [73]. Lastly, ECM density and tensional homeostasis has been shown to be regulated by miR-203 via the SLIT2/Robo1 receptor signalling pathway [74]. In mammary epithelial cells, repressed miR-203 expression enhances SLIT2/Robo1 signalling and the ability of mammary epithelial cell cytoskeleton to pull on collagen fiber [74]. 

## 6. The ECM in Lung Remodeling and Disease

**Figure 3:**
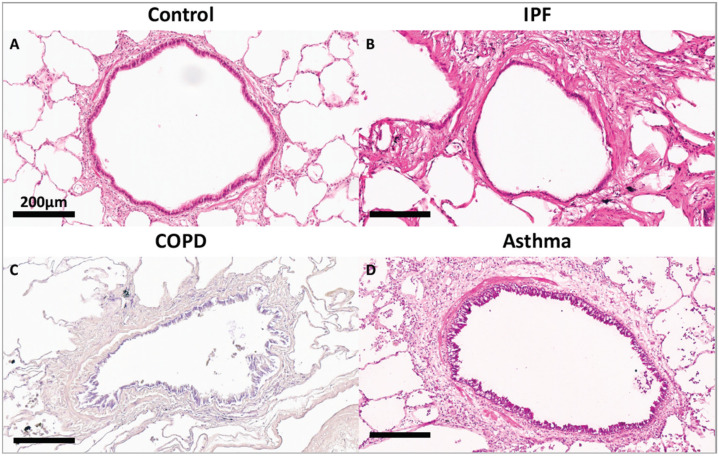
Representative image of a terminal bronchiole stained with hematoxylin and eosin from (**A**) donor control lung, (**B**) a lung from an end-stage idiopathic pulmonary fibrosis patient, (**C**) a lung from a patient with mild chronic obstructive pulmonary disease. (**D**) Terminal bronchiole stained with Masson’s trichome from a lung from a patient with asthma.

To maintain homeostasis, the lung ECM undergoes remodelling, a process whereby old or damaged ECM proteins undergo a series of proteolytic events and are replaced by newly synthesized proteins. Lung ECM remodeling is modulated by the synthesis rate of new ECM molecules and the surfeit of proteases released by specialized mesenchymal cells, predominantly fibroblasts and, to a lesser extent, airway smooth muscle, immune cells and epithelial cells of the lungs [4,75]. The remodelling process is directed by the interaction between the different cell types, cytokines, growth factors and enzymes within the lung ECM. The levels and ratios of ECM proteins during the repair response must be maintained to avoid disrupting the lung ECM characteristics, including tensile strength and elastic recoil. Upon lung injury, activation of the coagulation process initiates damage control and a provisional matrix (fibronectin), which is then followed by the development of an acute inflammatory response that recruits polymorphonuclear leukocytes (PMNs) and macrophages to protect against pathogens and remove debris from dead and dying cells. These important early steps prepare the site for activation of local fibroblasts, migrating myofibroblasts and recruited fibrocytes that remodel collagenous and noncollagenous tissues into scar tissue. Following a single injury, inflammatory cells usually leave the site before the resident fibroblasts and migrating myofibroblasts, and fibrocytes are activated to drive the tissue repair process. Over time, the provisional ECM is degraded via cell-mediated or proteolytic pathways and replaced with a restorative ECM consisting mainly of collagen I and collagen III, before these cells then undergo apoptosis [76]. In sharp contrast, repetitive injury is associated with the persistence of inflammatory immune cell infiltration during the remodelling of the ECM, which increases the potential for the development of an abnormal repair process creating further injury with abnormal scar formation, remodelling or permanent destruction of damaged tissue. Repeated lung injury and repair can trigger a vicious cycle of aberrant ECM protein deposition, accompanied by elevated ECM stiffness, further resulting in changes in cell phenotype and function, and this has a lasting effect on tissue function and ultimately disease progression [77]. Although the processes governing the resolution of injury repair are regulated by several pathways, in chronic fibrotic lung diseases the processes are compromised, thus resulting in impaired fibroblast proliferation, apoptosis and aberrant ECM remodelling [3,76]. Disruption of this balance changes the dynamics of the lung ECM with characteristics such as stiffness and elastance, as seen in asthma, chronic obstructive pulmonary disease (COPD) and idiopathic pulmonary fibrosis (IPF) [78,79,80]. Compared to the normal lung ECM (0.5–5 kPa), studies have shown that a stiff ECM (15–100 kPA) [81,82,83,84] can exacerbate TGF-β activation leading to enhanced pro-fibrotic signalling. Disruption of homeostasis within the lung ECM can also lead to the dysregulated synthesis of ECM proteins, such as fibrillar collagen I, by activated fibroblasts, leading to tissue fibrosis regardless of the inflammatory response [85]. The interplay between several diverse factors (cell types, genes, cytokines, growth factors, enzymes and epigenetics) is essential for the normal repair and remodeling processes within the lung [86]. Studies have shown that deviations in the posttranslational modification of ECM proteins, including enzymatic cross-linking, glycation and oxidation, impact the tensile strength, biomechanics and cell–ECM interactions and are contributors to lung diseases [87,88]. As shown in Figure 3, compared to a terminal bronchiole (last generation of conducting airways within the lung) from a donor control, there is extensive fibrosis surrounding the terminal bronchiole airway walls in the lungs from patients with IPF, COPD and asthma. Recent studies have provided evidence for the epigenetic role of miRNAs in the pathogenesis of lung diseases. For this review, only the regulatory effects of miRNA on ECM expression, degradation and topography in lung diseases, specifically, asthma, COPD and IPF, will be considered. 

### 6.1. Asthma

Asthma is characterized by chronic airway inflammation, bronchial hyperresponsiveness and airway remodeling. The deposition of ECM is a prominent feature of lung remodelling in patients with asthma irrespective of age, disease severity or steroid use [89,90,91]. ECM remodelling in asthma involves the airway epithelial basement membrane, laminar propria, bronchial and pulmonary vasculature [89,92,93]. The compositions of the airway smooth muscle (ASM) ECM, epithelial basement membrane and interstitial ECM have also been shown to be altered in asthma, with a predominant deposition of fibronectin, collagen I and III [94]. Most recently, the topography of fibrillar collagen fibers has also been assessed and shown to be more disorganized and fragmented in the airway laminar propria and pulmonary vasculature [89,95]. Several miRNAs have been reported to be differentially expressed in asthma and have primarily focused on the regulation of inflammation [96,97,98]. However, recent emerging studies using various samples including serum, ASM, bronchial fibroblasts and airway epithelial cells have shown miRNAs that are differentially expressed in asthma pathology and contribute to ECM remodelling. 

In addition to its role in bronchoconstriction, ASM plays an important interactive role with structural cells and inflammatory mediators contributing to inflammation and ECM remodeling in asthma [99]. In ASM cells from asthmatic patients, a number of miRNAs are downregulated compared to control subjects, leading to elevated ECM expression. The expression of miR-19 is decreased in ASM cells from asthmatic patients and induces elevated expression of collagen I, fibronectin and arginine methyltransferase activity through the ERK1/MAPK signalling pathway [100]. miR-204-5p has also been shown to be down-regulated in ASM cells from asthmatic patients and promotes the expressions of fibronectin and collagen III via the Six1 gene (a TGF-β1 inducible gene) [101]. Cheng and colleagues reported that miR-143-3p is significantly reduced in ASM cells of asthmatic patients compared to the controls [102]. They showed that miR-143-3p directly targets the nuclear factor of activated T-cells 1 (NFATc1) to promote collagen 1 and fibronectin expressions, leading to elevated ASM cell proliferation and up-regulation of CDK4 and cyclin D1 expressions. Lastly, Li and colleagues showed that miR-378 is elevated in ASM cells from asthmatic patients and, via MAPK and calcium signalling, can up-regulate collagen I and fibronectin expression [103].

With regards to inflammation and ASM, Liu and colleagues investigated the role of miR-145 in ASM cells treated with IL-1β, TNF-α and IFN-γ to mimic airway inflammation, and they found that miR-145 was significantly elevated and led to increased collagen I and myosin heavy chain expression through negative regulation of the transcription factor kruppel-like factor 4 (KLF4) protein and downstream activation of MMP-2 and MMP-9 [104]. Kuhn and colleagues showed that inhibition of miR-25 in IL-1β, TNF-α and IFN-γ-stimulated ASM cells, had a greater than two-fold down regulatory effect on collagen XI expression, and to a lesser extent the expressions of collagen (V and XV), fibronectin, MMP-9 and integrin (αm and β2), by stimulating KLF4 expression [105]. Growth factors such as TGF-β1 have also been shown to modulate miRNA expression in ASM. TGF-β1 has been shown to elevate miR-181a expression in ASM, leading to the overexpression of collagen I and fibronectin, via the Akt signalling pathway [106]. Lastly, miR-142 has been shown to be overexpressed in ASM cells derived from an asthma rat model and inhibits TGF-β expression via epidermal growth factor receptor (EGFR) signalling [107], leading to decreased collagen I and collagen III expressions. In support of this finding, the expression level of miR-142-3p was shown to be up-regulated in the sputum of asthmatic patients and correlates with sputum neutrophil cell counts [108].

In vivo models of allergic inflammation and asthma have shown that miR-485-3p is up-regulated in ASM cells derived from both pediatric and adult murine models of asthma [109]. miR-485-3p was shown to be involved in mediating airway remodelling by decreasing sprout-related EVH1 domain-containing protein (spred)-2 expression to promote growth factor-mediated Ras/ERK activation [109]. The overexpression of MiR-21-5p in an immunoglobulin E (IgE)-induced mouse model of allergic inflammation was found to increase the expressions of collagen I and fibronectin through inhibition of two signalling pathways: PTEN and PI3K/mTOR [110]. Furthermore, a recent study by Pan and colleagues using an ovalbumin-induced chronic murine model showed that the PI3K/Akt signalling pathway is regulated by increased miR-221 expression in ASM cells and leads to increased expressions of collagen I and collagen III [111]. 

Regarding other cell types within the lung, Zhang and colleagues showed that miR-221 expression is up-regulated in bronchial epithelial cells from asthmatic patients, and that overexpression of miR-221 significantly down-regulates sirtuin1 (SIRT1) expression in BEAS2B cells [112], which is known to regulate collagen I alpha-2 expression [113]. Yu and colleagues showed that TGF-β1-treated human bronchial fibroblasts have elevated miR-21, which leads to the up-regulation of collagen I alpha-1, fibronectin-1 and alpha-smooth muscle actin expressions, via negatively regulating SMAD7, leading to activated TGF-β1-SMAD signalling [114]. Lastly, treatment of the A549, BEAS-2B and H1299 cell lines with a miR-3162-3p mimic was shown to decrease β-catenin activation, leading to attenuation of TGF-β1-induced collagen I alpha-1 and fibronectin expressions [115]. In conclusion, there is strong evidence that alterations of miRNA signalling in asthma may lead to reorganization of the ECM particularly surrounding ASM. Further work is required to understand the contribution of miRNAs to ECM deposition in asthma, particularly in fibroblasts, epithelial and endothelial cells.

### 6.2. Chronic Obstructive Pulmonary Disease

COPD is characterized by chronic lung inflammation and irreversible airflow limitation. COPD is caused by the chronic inhalation of cigarette smoke or other harmful particles, which cause pulmonary injury, leading to chronic airway inflammation and tissue remodelling. The major histopathological changes observed within the lung include chronic bronchitis, small airway disease and emphysema. Recent studies have shown that small airway disease precedes emphysematous tissue destruction, suggesting a temporal pattern of ECM deposition and destruction in the disease progression [116]. Using a large cross-sectional study, Hogg and colleagues showed that the progression of COPD from GOLD (Global Initiative for Obstructive Lung Disease) stage 0 to GOLD stage 4 is strongly associated with thickening of the airway wall and each of its compartments by remodelling of the ECM, which affects airway wall elasticity, thickness and resistance [117,118]. In addition, loss of elastic recoil from the destruction of the ECM within the parenchyma (emphysematous tissue destruction) is a well-described feature of COPD [119], with several studies showing decreased protein expression and volume fraction of elastin in both the conducting airways and the parenchyma of patients with mild, moderate or severe COPD [120,121].

With regards to miRNAs, several studies have also shown changes in the epigenetics of the COPD lung and implicated miRNAs as an important player in COPD pathology [122,123,124,125]. When looking at ECM remodelling in COPD lung tissue, miR-15b has been shown to be significantly increased in lung tissue from patients with mild, moderate or very severe COPD, with the highest expression in very severe cases [125]. The same study also showed that in the epithelial BEAS2B cell line, TGF-β treatment leads to increased expression of miR-15b, which negatively regulates decorin expression via SMAD7 and SMURF2. This finding correlates with the results obtained by Noordhoek and colleagues, who showed decreased expression of decorin in fibroblasts obtained from the lung tissue of patients with mild or severe COPD [126]. Dang and colleagues (2019) have also reported a decreased expression of miR-145-5p in the lung tissue of smokers with mild to moderate COPD compared to non-smokers [127]. Furthermore, the authors showed that human bronchial epithelial cells (HBECs) when exposed to cigarette smoke extract (CSE), down-regulate miR-145-5p expression, leading to increased Kruppel-like factor subfamily-5 (KLF5) expression level. Abe and colleagues (2016) have previously reported that KLF5 is up-regulated in fibroblasts and endothelial cells from ex-smokers with COPD, which is correlated with the severity of airflow limitation in patients with COPD [128]. The authors also showed that silencing of KLF5 suppressed oxidative/nitrosative stress-related responses within lung fibroblasts, leading to decreased collagen, MMP-2 and MMP-9 expression. Lastly, Du and colleagues (2017) found decreased expression levels of the miR-181c in parenchymal tissue of patients with very severe COPD and CSE-exposed HBECs [129]. The miR-181c was shown to negatively regulate the transcription factor CCN1 (Cellular Communication Network Factor 1) to induce TGF-β-mediated fibronectin and collagen up-regulation via SMAD protein phosphorylation [129,130]. 

In terms of fibroblasts, which are an important producer of ECM within the lamina propria, Ong and colleagues showed that parenchymal lung fibroblasts of COPD patients overexpress miR-455-3p and miR-21-3p, compared to controls, when stimulated by TGF-β, resulting in the induction of fibronectin and collagen I through the TGF-β and wnt (wingless and Int-1) signalling pathways [131]. 

In terms of in vivo models, Chi et al. (2019) and Tang et al. (2019) in a rat model of COPD showed decreased expression of miR-29 [132,133]. Using rat airway epithelial cells from the model, they found that the decrease in miR-29 expression was found to correlate with the increased expression level of collagen III alpha-1 and collagen IV alpha 1 [134]. In summary, a number of studies have demonstrated miRNAs regulating ECM synthesis, primarily collagen and fibronectin, by epithelial cells and fibroblasts in patients with COPD in mild to very severe disease.

### 6.3. Idiopathic Pulmonary Fibrosis

IPF is the most common and progressive type of idiopathic interstitial pneumonia that is unresponsive to treatment, leading to a median survival of 3–5 years [135,136,137,138]. The pathology of IPF is characterized by heterogeneous interstitial fibrosis, honeycomb cysts and fibrotic foci associated with excessive deposition of ECM proteins resulting in aberrant matrix metalloproteinases, connective tissues, morphogens and impaired signalling of growth factors [139,140,141,142,143]. The current hypothesis for tissue destruction in IPF involves continuous damage and senescence of the alveolar epithelium, leading to the destruction of the basement membrane and activation of myofibroblasts [144]. As the disease progresses, the composition of the ECM has been shown to vary, with an accumulation of versican in the onset of IPF, whereas collagen I and collagen III accumulation is observed in both the early and late stages of IPF [145]. The severity of fibrosis in IPF has also been shown to correlate with the number of elastic fibers, with a higher elastic fiber score being related to worse disease outcomes [146]. The diseased ECM is a critical linchpin in IPF, and it serves as a causal link for alteration in cell gene expression patterns at the translational level [3]. Several studies have implicated the role of miRNAs in IPF pathology [139,147,148]. 

Recently, Guiot and colleagues (2020) showed that the expression levels of miR-142-3p are elevated in sputum and plasma exosomes of IPF patients and positively correlate with sputum macrophage number [149]. This group further showed that miR-142-3p represses TGFβ-R1 in both A549 and a fetal lung fibroblast cell line (MRC5), resulting in decreased collagen I alpha-1 and collagen III alpha-1 expression. In bronchoalveolar lavage cells of IPF patients, miR-29a and miR-185 are down-regulated, leading to activation of TGF-β and PTEN signalling and elevated collagen I alpha-1 expression [150]. Within the IPF lung, the expression level of miR-200 is decreased and was shown to correlate with collagen I alpha-1 and fibronectin expression. However, Yang and colleagues showed that the miR-200 expression level is higher in airway epithelial cells compared to lung fibroblasts in mice with experimental pulmonary fibrosis [151]. Specifically, in the alveoli epithelium, IPF patients compared to donor controls have decreased expression of let-7d. Pandit V. et al (2010) showed reduced expression of let-7d, which led to overexpression of its direct target HMGA2 (high-motility group AT-hook-2), resulting in the over-expression of mesenchymal markers, decreased expression of epithelial markers and increased collagen deposition in alveolar epithelial cells [152].

In vivo models using bleomycin are commonly used to understand inflammation and ECM remodeling in IPF. In the lung of bleomycin-treated mice, miR-26 and miR-326 are down-regulated, leading to increased collagen synthesis via TGF-β1/Smad signalling [153,154]. Down-regulation of miR-15a expression in bleomycin-treated mice has also been shown to increase the expressions of collagen I alpha-1, collagen II alpha-1, fibronectin 1, alpha-SMA and connective tissue growth factor (CTGF) via the yes-associated protein (YAP)1/twist/TEAD dependent pathway [155]. In Lung fibroblasts derived from bleomycin-induced mice compared to controls, there is down-regulation of miR-29 expression [156]. Xiao and colleagues (2012) further showed that miR-29 is negatively regulated by the TGF-β/Smad pathway, altering ECM remodelling by promoting the expression of fibronectin, collagen I and collagen III [156]. In agreement with this finding, Cushing and colleagues (2011) showed in the fetal lung fibroblast cell line (IMR-90) that knock-down of endogenous miR-29 leads to upregulated expression of collagen types (I, III, IV, V, XV) and laminin (alpha-1,3, beta-1) via TGF-β1 and possibly NF-kB signalling pathways [134]. Lung myofibroblasts isolated from bleomycin-treated mice have elevated miR-21 levels, which correlated with fibronectin lung expression. miR-21 was shown to stimulate Smad7 and PTEN/ERK signalling, and treatment of myofibroblast cells with miR-21 antisense probes prevented the enhanced deposition and synthesis of collagen and fibronectin [157]. In bleomycin-treated pleural mesothelial cells (PMC), miR-18a-5p expression is decreased, which is a direct target of TGF-β receptor II [158]. Decreased miR-18a-5p led to over-expression of alpha-SMA, collagen I and vimentin while decreasing E-cadherin and cytokeratin 8 expressions [158]. In summary, a number of studies have demonstrated miRNAs regulating ECM synthesis, primarily collagen, through TGF-β signalling fibroblasts in patients with IPF.

## 7. Potential for miRNA Therapeutics in Lung Disease

Compared to disease conditions with a single genetic link, we now understand that complex lung diseases such as asthma, COPD and IPF involve multiple genes and pathways. As miRNAs can target hundreds and potentially thousands of genes, miRNAs may prove therapeutically advantageous to regulate entire biological pathways that are pathogenically altered in complex diseases. It should be noted that the pleiotropic nature of miRNAs does warrant concern for off-target effects; however, steroids commonly used in multiple inflammatory diseases are effective due to their pleiotropic effects and provide further support for miRNAs as future therapeutics. 

As miRNAs are ubiquitously expressed throughout the body, they can readily be measured from peripheral blood, tissue biopsies, saliva, urine, cerebrospinal fluid (CSF) and other biological samples [53,159,160]. Due to this accessibility, a growing number of studies have shown that one or subsets of miRNAs can be used as biomarkers to indicate disease pathology, staging and progression with high sensitivity and specificity [161,162,163,164,165,166,167,168,169,170,171]. To date, several miRNA biomarker clinical studies have been completed in the clinicaltrials.gov database for a range of conditions including breast cancer, coronary heart disease, diabetes, epilepsy, influenza and stroke.

In terms of use as therapeutic interventions, the first small-interfering RNA (siRNA) drug trial in humans was conducted in 2004. Fifteen years later, the drug Patisiran was granted FDA approval in 2018 for treatment of polyneuropathy caused by hereditary transthyretin-mediated (hATTR) amyloidosis. While there are no current FDA-approved miRNA therapeutics for medical intervention, several miRNA drug candidates have been entered into the clinicaltrials.gov database as phase 1 or 2 clinical trials for a range of health conditions including cancer, hepatitis C, heart abnormalities, kidney disease and pathologic fibrosis. Relevant to this review, there is a current phase 2 clinical trial of Remlarsen (mir-29) from Mirage Therapeutics, which is focused on decreasing the expression of collagen and other proteins involved in scar formation over a 1 year follow up [172]. This study will be of great interest to understand the potential of miRNAs as therapeutics to modify the pathogenic remodelling of the ECM in the skin and other organs such as the lung.

In terms of administration, current miRNA trials are focused on injection or intravenous administration to mimic (over-express transcript) or silence (repress transcript) mRNA. For cancer treatments, injection into the tumour site has been shown to enhance target specificity, efficacy and minimise side effects [173,174]. Another recent application of miRNAs is focused on improving drug resistance. A number of studies have shown that manipulating the expression of specific miRNAs can alter drug sensitivity. For example, many of the standard therapies for breast cancer, such as doxorubicin, cisplatin, and Taxol, are all associated with deregulated miRNAs, which may modulate resistance to the drug [170]. The altered expression of miRNAs has also been associated with drug resistance in the treatment of conditions such as epilepsy [163], multidrug-resistant (MDR) tuberculosis [175] and insulin sensitivity [176,177]. Emerging evidence suggests that the ATP binding cassette (ABC) transporter family of proteins that is involved in drug resistance is regulated by miRNAs, and this provides a potential mechanism for the use of miRNAs in drug resistance [178].

## 8. Conclusions

The initiation and progression of chronic lung diseases are modulated by complex environmental and epigenetic factors. In the initiation and progression of these diseases, there is an intricate interaction between cells and the ECM through various molecules and signalling pathways, amongst which epigenetic regulation by miRNAs has emerged as an important player. We have outlined in this review the current knowledge on miRNAs regulating ECM synthesis, degradation and topography by cells and their dysregulation in asthma, COPD and IPF. The accumulating evidence of differences in the expression profiles of miRNAs in lung diseases, and their correlation with lung function measures and ECM gene targets, highlights the potential of miRNAs as biomarkers for diagnosis, staging and future therapeutic drugs in chronic lung disease.

## Figures and Tables

**Table 1:** Regulation extracellular matrix remodeling by miRNAs.

ECM-Related Molecule	miRNA	Direction of miRNA Change	Direction of ECM Molecule Change	Mechanism	Cell Type	Disease/Develop-Mental PROCESS	Reference
**ECM Proteins**							
Collagen I	miR-205	↑	↓	binds to the ∆Np63	cardiomyocytes	myocardial fibrosis	Castoldi et al., 2011
Collagen I	miR-133a, miR-29b	↑	↓	bind to COL1A1 3’UTR	Hepatic stellate cells	liver fibrosis	Sekiya et al., 2011
Collagen I	miR-29b	↑	↓	Targets COL1A1, COL1A2, ITGB1, and PDGFR-B	Hepatocellular carcinoma cells	hepatocellular carcinoma	Ji et al., 2010
Collagen I	Let-7g	↑	↓	Let-7g binds to COLIA1 3’UTR	Hepatocellular carcinoma cells	hepatocellular carcinoma	Ji et al., 2011
Collagen I	miR-21, miR-29a	↑ (μιΡ21), ↓ (μPNA-29α)	↓ (μιΡ21), ↓ (μPNA-29α)	targets TGF-β signalling pathway	dermal fibroblasts	systemic sclerosis	Jafarinejad-Farsangi et al., 2019
Collagen I	miR-152	↑	↓	binds to 3’UTR of ITGA5	human dermal fibroblasts	Ageing	Mancini et al., 2012
Collagen III	miR-29	↓	↑	-	leiomyoma and myometrial cells	uterine fibroids	Marsh et al., 2016
Collagen IV	miR-29	↑	↓	binds to 3’UTR		Ageing	Takahashi et al., 2012
Collagen V	miR-185, miR-186	↓	↑	binds 3’UTR of COL5A1	human alveolar epithelial cells	Idiopathic pulmonary fibrosis	Lei et al., 2016
Fibronectin type III domain containing 3B	miR-143	↑	↓	binds to FNDC3B 3’UTR	hepatocytes	hepatocellular carcinoma	Zhang et al., 2009
Fibronectin	miR-146a	↓	↑	binds to fibronectin 3’UTR	renal endothelial cells	Chronic diabetes	Feng et al., 2011
Fibronectin	miR-377	↑	↑	taregts p21-activated kinase and superoxide dismutase expression	mesangial cells	Diabetic nephropathy	Wang et al., 2008
Fibronectin I	miR-206	↓	↑	binds 3’UTR of fibronectin 1	human lung adenocarcinoma epithelial cells	bronchopulmonary dysplasia	Zhang et al., 2013
Fibronectin	miR-17	↑	↓	binds 3’UTR of fibronectin	endothelial cells	Health	Shan et al., 2009
Laminin and integrin	miR-29s	↑	↓	binds 3’UTR of LAMC2 and ITGA6	head and neck squamous carcinoma cells	Cancer	Kinoshita et al., 2013
Laminin-322	miR-218	↓	↑	binds to 3’UTR of LAMB3	head and neck squamous carcinoma cells	Cancer	Kinoshita et al., 2012
SPARC, Collagen I, Collagen IV, laminin	miR-29	↑	↓	Targets TGF-β2/SMAD3 signalling	human TM cells	Glaucoma	Villarreal et al., 2011
Collagen XVI	miR-181a	↑	↓	binds to 3’UTR of COL16A1	human dermal fibroblasts	Ageing	Mancini et al., 2012
Laminin γ1 (LAMC1)	miR-205	↓	↑	binds 3’UTR of LAMC1	triple-negative breast cancer cells	breast cancer	Piovan et al., 2012
Laminin γ1 (LAMC1)	miR-124a	↓	↑	binds 3’UTR of LAMC1	Glioblastoma cells	Glioblastoma (GBM)	Fowler et al., 2011
laminin β2 (LAMB2)	Let-7b	↑	↓	down-regulates transcription factor HMGA	differentiating podocytes	Diabetic nephropathy	Schaeffer et al., 2012
Perlecan/Heparin sulfate proteoglycan 2	miR-663	↑	↓	binds 3’UTR of HSPG2	chemoresistant breast tumour cells	breast cancer	Hu et al., 2013
Syndecan-1	miR-10b	↑	↓	binds 3’UTR of syndecan 1	breast cancer cells	breast cancer	Ibrahim et al., 2012
Glypican-3 and fibronectin-1	miR-96	↑	↓	binds 3’UTR of glypican-3 and fibronectin-1	hepatocytes	hepatocellular carcinoma and hepatoblastoma	Jalvy-Delvaille et al., 2011
Nephronectin	miR-378	↑	↑	binds 3’UTR of nephronectin	osteoblasts	bone development	Kahai et al., 2009
Nephronectin	miR-23a, miR-101a, miR-296-5p, miR-328, miR-340-3p, miR-425	↑	↑	binds 3’UTR of nephronectin	osteoblasts	Bone transplant/repair	Lee et al., 2011
Versican	miR-138	↑	↓	binds to CSPG2 3’UTR	cardiomyocytes	disrupted cardio-morphogenesis	Morton et al., 2008
Versican	miR-143	↓	↓	binds to 3’UTR of versican	cardiomyocytes	Cardiovascular diseases	Wang et al., 2010
Aggrecan	miR-181a	↑	↓	targets *CCN1* and *ACAN* signalling	human chondrocytes	cartilage metabolism	Sumiyoshi et al., 2010, Sumiyoshi et al., 2013
Glypican-3	miR-1291, miR-1271	↓	↑	binds to 3’UTR of GPC3 and IRE1α	hepatocytes	hepatocellular carcinoma	Maurel et al., 2012, Maurel et al., 2013
Glypican-3	miR-219-5p	↓	↑	binds to 3’UTR of GPC3	hepatocytes	hepatocellular carcinoma	Huang et al., 2012
Glypican-3	miR-520c-3p	↓	↑	binds to 3’UTR of GPC3	hepatocytes	hepatocellular carcinoma	Miao et al., 2014
Glypican-4	miR-125a	↑	↓	binds to 3’UTR of glypican-4	hepatocytes	cell proliferation	Feng et al., 2012
**ECM enzymes**							
MMP-1	miR-203	↑	↑	-	synovial fibroblasts	rheumatoid arthritis	Stanczyk et al., 2011
MMP-2	miR-29b	↑	↓	binds 3’UTR of COL1A1, COL3A1, COL4A1, and MBP-1	prostate cancer cells	prostate cancer	Steele et al., 2010
MMP-2, MMP-9	miR-145	↑	↓	binds KLF5 3’UTR	airway smooth muscle cells	Asthma	Liu et al., 2015
MMP-2, MMP-9	miR-206	↓	↑	Targets Rho-cdc42/myosin signalling	Epithelial breast cancer cell line (MDA-MB-231)	Breast cancer	Liu et al., 2018
MMP-2, MMP-9	miR-340	↓	↑	binds to 3’UTR of C’Met	breast cancer cells	Breast cancer	Wu et al., 2011
MMP-3	miR-155	↑	↓	targets LPS signalling, TNF-receptor superfamily-interacting serine-threonine kinase 1 and IkB kinase activity	synovial fibroblasts	rheumatoid arthritis	Stanczyk et al., 2008
MMP-9	miR-218	↑	↑	binds to MMP-9 3’UTR	Rat osteoclasts	Periodontitis	Guo et al., 2018
MMP-9	MiR-373	↑	↑	binds to the 3’UTR of mTOR and SIRT1	Human fibrosarcoma cells	cancer	Liu P. and Wilson M., 2011
MMP-9	miR-340	↓	↑	binds to MMP-9 3’UTR	retinal ganglion cells	Glaucoma	Surgucheva et al., 2010
MMP-9	miR-212, miR-132	↑	↓	binds to MMP-9 3’UTR	*miR-212/132*^−/−^ mice	mammary gland development	Ucar et al., 2010
MMP-13	miR-27b	↓	↑	binds to MMP-13 3’UTR	chondrocytes	osteoarthritis	Akhtar et al., 2011
MMP-13	miR-143	↓	↑	-	osteosarcoma cells	osteosarcoma	Osaki et al., 2011
MMP-14	let-7	↓	↑	Targets TGF-β1/ERK signalling	pancreatic cancer cells	pancreatic ductal adenocarcinoma	Dangi-Garimella et al., 2011
MMP-16	miR-146b	↑	↓	binds to MMP-16 3’UTR	glioblastoma cells	glioblastoma	Xia et al., 2009
MMP-16	miR-155	↑	↓	binds to MMP-16 3’UTR	cardiomyocyte progenitor cells	Transplantation therapy	Liu et al., 2012
TIMP-1	miR-29a	↑	↓	binds to TAB1 3’UTR	dermal fibroblasts	systemic sclerosis	Ciechomska et al., 2014
RECK	miR-21	↑	↓	binds to 3’UTR of RECK	glioma cells	glioblastoma	Gabriely et al., 2008
TIMP-3	miR-181b	↑	↓	Targets miR-191b	Hepatocellular carcinoma cells	Hepatic cancer	Wang et al., 2010
TIMP-3	miR-221, miR-222	↑	↓	bind to 3’UTR of TIMP3	Non-small cell lung cancer and hepatocarcinoma cells	small cell lung cancer and hepatocellular carcinoma	Garofalo et al., 2010
TIMP-3	miR-181a	↑	↓	-	osteosarcoma cells	osteosarcoma	Jianwei et al., 2013
Neutrophil elastase(HNE), MUC5ac, EGFR	miR-146a	↓	↑	binds to EGFR 3’UTR	human bronchial epithelial cells	Mucus hypersecretion	Zhong et al., 2011
Heparanase (HPSE)	miR-30	↑	↓	Targets TGF-β1 and IL-6 signalling	human melanoma cells	melanoma	Liu et al., 2013
Heparanase (HPSE)	miR-1258	↑	↓	binds to HPSE 3’UTR	BMBC cells	brain metastatic breast cancer	Zhang et al., 2011
Heparanase (HPSE)	miR-1252-5p	↓	↑	binds to HPSE 3’UTR	Multiple myeloma cells	Multiple myeloma	Rodrigues et al., 2021
**Cytokines and growth factors**							
IL-6	miR-203	↑	↑	-	synovial fibroblasts	rheumatoid arthritis	Stanczyk et al., 2011
Keratinocyte growth factor	miR-155	↑	↓	binds to 3’UTR of KGF	lung fibroblasts	IPF	Pottier et al., 2009
TGF-β1	miR-26a	↑	↓	binds to 3’UTR of HMGA2	Lung epithelial cells	IPF	Liang et al., 2014
TGF-β	miR-200	↓	↑	Targets TGF-β/bone morphogenetic protein signalling	Triple-negative breast cancer cell	Breast cancer	Truong et al., 2014
TGF- β	miR-29b	↓	↑	binds to 3’UTR of HSP47	Skin fibroblast	Wound healing	Zhu Y. et al, 2016.
TGFBR2	miR-153	↓	↓	binds to TGFBR2 3’UTR	lung fibroblasts	IPF	Liang et al., 2015
TGFBR2	miR-145	↑	↓	binds to 3’UTR of TGFBR2	Aortic smooth muscle cell	Vascular diseases	Zhao et al., 2016
TGFBR1, CTGF, COL1A1	miR-133a	↑	↓	binds to 3’UTR of TGFBR1, CTGF and COL1A1	human lung fibroblasts	IPF	Wei et al., 2019
VEGF	miR-503	↓	↑	miR-503 binds to VEGF 3’ UTR	human lung fibroblasts	COPD	Ikari et al., 2017
VEGF-A	miR-126	↓	↑	miR-126 binds to VEGF-A 3’ UTR	Oral squamous cell carcinoma cell	oral cancer	Sasahira et al., 2012
VEGFA/CCL2, FOXO3a	miR-1, miR-31, miR-206	↓	↓	bind to 3’UTR of CCL2/VEGFA and FOXO3a	cancer-associated lung fibroblasts	lung cancer	Shen et al., 2016
FOXO3a	miR-96	↓	↑	binds to FoxO3a 3’UTR	human lung fibroblasts	IPF	Nho et al., 2014
FGF2	miR-195	↓	↑	binds to FGF2 3’UTR	prostatic cancer cells	prostate cancer	Liu et al., 2015
Smad7	miR-21	↑	↑	binds to Smad7 3’UTR	myofibroblasts	IPF	Liu et al., 2010
SLIT2/Robo1	miR-203	↓	↑	binds to Robo1 3’UTR	mouse mammary gland cells	breast tumour	Le et al., 2016
**Topography**							
Collagen	miR-203	↓	↑	targets SLIT2/Robo1 signalling	Mammalian epithelial cell	Breast tumour	Le et al., 2016
LOX	miR-19b	↓	↑	targets CTGF	myocardium	Aortic stenosis	Beaumont J. et al., 2017
LOXL2	miR-29a, miR-29b, miR-29c	↓	↑	MiR-29 family directly target LOXL2	Renal cell carcinoma	Renal cell carcinoma	Nishikawa R. et al., 2015
LOXL2, PLOD2	miR-26a, miR-26b	↓	↑	bind to 3’UTR of LOXL2 and PLOD2	renal cell carcinoma cells	renal cell carcinoma	Kurozumi et al., 2016
LOX, LOX1, elastin and collagen	miR-145	↓	↑	targets notch signalling	Vascular muscle cells	Angiotensin II-induced fibrosis	Zhao N. et al., 2015

## Data Availability

Not applicable.
